# Distal Upper Molar Force Distribution With Clear Aligners Using Different Anterior Teeth Anchorage Setups: A Finite Element Study

**DOI:** 10.1111/ocr.70077

**Published:** 2025-12-13

**Authors:** Weber Jose da Silva Ursi, Yamyle Claudia Velásquez Barragán, Ki Beom Kim, Carlos Flores‐Mir, Guilherme de Araujo Almeida

**Affiliations:** ^1^ Department of Social and Clinical Pediatric Dentistry Dental School of São Paulo State University São José dos Campos São Paulo Brazil; ^2^ Post‐Graduate Program of Dental School Federal University of Uberlandia Uberlandia Minas Gerais Brazil; ^3^ Dr. Lysle Johnston Endowed Chair in Orthodontics and the Program Director in the Orthodontic Department, Center for Advanced Dental Education Saint Louis University Saint Louis Missouri USA; ^4^ Department of Dentistry and Dental Hygiene University of Alberta Edmonton Canada; ^5^ Department of Pediatric Dentistry and Orthodontics Dental School of Federal University of Uberlandia Uberlandia Minas Gerais Brazil

**Keywords:** clear aligners, finite element analysis, molar distalization, skeletal anchorage

## Abstract

**Objective:**

The notion that clear aligners alone can distalize upper molars without affecting anterior teeth is inaccurate. Although strategies such as Class II elastics, tooth‐movement sequencing, and attachment variations have been investigated to mitigate unwanted side effects, temporary anchorage devices have demonstrated potential for maintaining anchorage during molar distalization. This study used Finite Element Analysis to evaluate different setups for distalizing one or both upper molars, comparing passive anchorage (ligature tie) and active anchorage (1.66 N), and assessing the presence of vertical attachments.

**Materials and Methods:**

Six models were generated with 0.2 mm distal activation for molar distalization. These models varied by premolar/M attachments and anchorage type—active (1.66 N) or passive—applied from extra‐alveolar screws to canine buttons.

**Results:**

All setups distalized the second molars, but passive anchorage demonstrated greater efficiency and fewer side effects. Passive systems achieved over 90% distalization‐to‐anchorage loss ratios, compared with 65% with active forces. Passive setups also minimised unintended anterior movement and enabled distal canine movement following molars—an advantageous outcome. Vertical attachments had minimal impact. *X*‐axis (midline) movement predominantly affected canines, particularly with active anchorage. Anterior intrusion on the *Z*‐axis was reduced with passive systems.

**Conclusion:**

Active anchorage forces may deform aligners and compromise control, whereas passive anchorage—similar to a ligature wire applied to anterior teeth—supports planned movement without disrupting biomechanics.

## 1 Introduction

Anterior teeth anchorage control is essential for successful orthodontic molar distalization. Although clear aligner setups provide moderate anchorage, they often result in some loss of anterior anchorage during distalization without additional support [1]. To enhance anchorage and reduce unwanted anterior movement or tipping, skeletal anchorage devices—such as miniscrews or temporary anchorage devices (TADs)—have been effectively combined with clear aligners [2]. Previous research has shown that integrating skeletal anchorage with aligners improves posterior tooth movement control and preserves anterior tooth position, thereby reducing anchorage loss [3]. This approach is particularly beneficial in non‐extraction cases that require space creation through distalization without compromising anterior occlusion or aesthetics.

Clinicians are increasingly using miniscrews, which can be placed by orthodontists in buccal or palatal regions, between or beside tooth roots [4, 5, 6, 7]. In the upper jaw, the infrazygomatic crest is a preferred and stable site for screw placement because it typically does not require repositioning during forward or backward movement of tooth segments [6, 8]. Anchorage can be applied to aligners via precision cuts, bonded buttons, or power arms of varying lengths [9, 10]. This anchorage support is commonly delivered using elastic bands, chains, or closed‐coil springs.

However, questions remain regarding the biomechanical effectiveness of these methods. Should miniscrew support solely provide anchorage (passive anchorage), or should it also actively contribute to distal tooth movement (active anchorage)? In passive anchorage, support is delivered via a ligature wire to an anchoring tooth (typically a premolar or canine) that counteracts the forward force generated by distal activation of posterior teeth. In contrast, active anchorage applies a distalizing elastic force directly to the anterior segment using a bonded button, precision cut, or power arm—serving both as anchorage and as an additional source of distalizing force [7, 9, 11, 12, 13].

To investigate these factors, this study employed finite element analysis (FEA) to evaluate the force system generated by distalizing the upper first and/or second molars. Two anchorage strategies were analyzed: (1) active anchorage force (1.66 N) and (2) passive anchorage simulating ligature wires from an extra‐radicular miniscrew in the infrazygomatic crest to bonded buttons on the upper canines. The biomechanical effect of adding vertical rectangular attachments to the virtual setup was also assessed.

## 2 Materials and Method

This study received prior approval from the Research Ethics Committee of the Federal University of Uberlandia (UFU), Brazil, and the National Research Ethics Council (CONEP—Brazil) (CAAE: 68334822.0.0000.5152).

A cone‐beam computed tomography (CBCT) scan of an 18‐year‐old Caucasian female with Class II, Division 1 malocclusion, healthy periodontium, and a full complement of permanent teeth (excluding third molars) was used. Pre‐treatment records, stored as image files at the Dental School of UFU (FOUFU), were utilised with informed consent.

The CBCT images were acquired using a Classic i‐CAT tomograph (90 kV, 15 mA, 10.5 cm × 13.0 cm field of view, 0.18 mm slice thickness, 0.3 mm voxel dimension). The effective radiation dose was 125 μSv, and the scan duration was 4.5 s.

The upper arch from the selected tomographic image, in DICOM format, was imported into InVesalius CTI software (Renato Archer, Campinas, São Paulo, Brazil) to delineate anatomical boundaries, which were then saved as STL (stereolithographic) files. For this purpose, the upper jaw was segmented from the incisal edges of the teeth to the level of the zygomatic bone. Based on image density, the following maxillary structures were delineated: compact bone, trabecular bone, enamel, dentine, pulp, and the periodontal ligament [14, 15]. A 0.2 mm thick periodontal ligament layer [16] was created around each tooth root using Boolean operations [14].

Following segmentation, a three‐dimensional (3D) triangular surface model of each maxillary structure was exported in STL format. Using 3‐matic software (version 18.0; Materialise, Leuven, Belgium), the aligner, bonded buttons, and precisely dimensioned vertical rectangular attachments (not bevelled) were designed. An extra‐alveolar screw (STL), supplied by Peclab (extra‐alveolar mini‐implant 5593; 12 × 2 mm), Peclab, Belo Horizonte, Brazil, was placed on the zygomatic crest. Its position was 11 mm superior to the mesiobuccal cusp of the upper second molar, with a 70° angulation relative to the occlusal plane [8]. The aligner material was modelled as 0.75 mm thick and custom‐fit to the crowns of the upper teeth. Bonded buttons were positioned at the middle of the cervical region of the upper canines. The STL surface files were then imported into Femap software (Siemens PLM Software, Plano, Texas, USA) to generate 10‐node tetrahedral meshes for volumetric element formation. Model convergence was verified through mesh refinement, particularly at contact interfaces, geometric discontinuities, and in the upper molars, canines, and incisors. The refinement process was continued until the results became independent of the number of elements.

**FIGURE 1:**
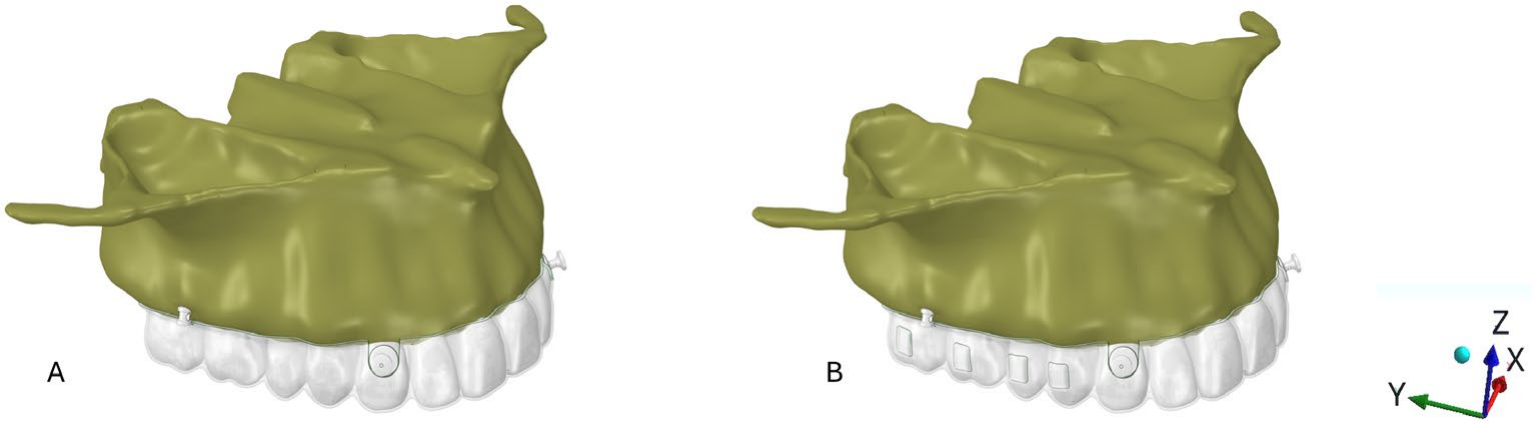
(A) Models 1–3 (without attachments) and (B) Models 4–6 (with vertical attachments on molars and premolars). Active anchorage (1.66 N) was applied in Models 1, 2, 4, and 5, whereas passive anchorage (0.012‐in. stainless steel ligatures) was applied in Models 3 and 6. Anchorage was delivered from extra‐alveolar screws to bonded buttons in all models. The representation of the *x*‐, *y*‐, and *z*‐axes is also shown.

Subsequently, all described structures were exported to SolidWorks CAD software (Dassault Systems, Paris, France) to create the study models. Six models were generated, varying according to the presence or absence of non‐bevelled vertical rectangular attachments on premolars and molars and the application of anchorage force. FEA was conducted under static loading [5]. The step distance for molar distalization was set at 0.2 mm of displacement along the positive *y*‐axis of the original model. A clear aligner was then constructed under these loading conditions, and the loading force was applied due to the mismatch between the new aligner and the original model. The same procedure was repeated for the simultaneous distalization of both molars, with an additional 0.2 mm displacement applied between both molars and between the first molar and the second premolar (Figure 1). Specifically, either an active anchorage force (1.66 N) or passive anchorage was applied from the bonded buttons positioned in the middle of the cervical region of the canines to the extra‐alveolar screws. The active anchorage force of 1.66 N was applied to the nodes of the button, directed toward the extra‐alveolar screw. For the passive simulation, a restriction was applied to the movements of the button nodes, preventing displacement along the *y*‐axis in the mesial (negative) direction.

### 2.1 Group I

Model 1: No attachments; 0.2 mm distal activation between the first and second molars; active anchorage (1.66 N) from extra‐alveolar screws to bonded buttons.

Model 2: No attachments; 0.2 mm distal activation between the molars and second premolars; active anchorage (1.66 N) from extra‐alveolar screws to bonded buttons.

Model 3: No attachments; 0.2 mm distal activation between the molars and second premolars; passive anchorage (0.012‐in. stainless steel ligature) from extra‐alveolar screws to bonded buttons.

### 2.2 Group II

Model 4: Vertical attachments on molars and premolars; 0.2 mm distal activation between the first and second molars; active anchorage (1.66 N) from extra‐alveolar screws to bonded buttons.

Model 5: Vertical attachments on molars and premolars; 0.2 mm distal activation between the molars and premolars; active anchorage (1.66 N) from extra‐alveolar screws to bonded buttons.

Model 6: Vertical attachments on molars and premolars; 0.2 mm distal activation between the molars and premolars; passive anchorage (0.012‐in. stainless steel ligature) from extra‐alveolar screws to bonded buttons.

For structural analysis, each model's mesh was imported into ANSYS software (ANSYS Inc., Canonsburg, Pennsylvania, USA). Smooth contact was defined between the alveolar bone and extra‐alveolar screws. Attachments and bonded buttons were modelled as rigid bodies. Nodes on the maxilla—excluding the palate—were fully constrained in the *x*‐, *y*‐, and *z*‐directions (Figure 1). A friction coefficient of 0.2 was applied between the dental crowns and aligners. All materials were modelled as linear‐elastic, isotropic, and homogeneous, with Poisson's ratios and elastic moduli listed in Table 1 [14, 15, 16, 17, 18]. Model validation involved a two‐step process. First, coherence analysis was performed to confirm the expected stress direction and distribution. Second, the initial scan of the maxilla was superimposed onto a subsequent scan that included a 0.2 mm interproximal separator between the maxillary molars using the palatal rugae as reference [19]. The displacement of the second molar, canine, and upper central incisor was then measured and compared with the finite element model results.

**TABLE 1:** Mechanical properties used for orthotropic and isotropic structures.

Structures	Longitudinal	Orthotropic structures	*Z*
		Elastic modulus (MPa)	
		Transverse	
Enamel	73,720	63,270	63,270
Dentine	17,070	5610	5610
		**Shear coefficient (MPa)**	
Enamel	20,890	24,070	20,890
Dentine	1700	6000	1700
		**Poisson ratio (v)**	
Enamel	0.23	0.45	0.23
Dentine	0.3	0.33	0.3

Structures	Isotropic structures	
	Elastic modulus (MPa)	Poisson ratio (v)
Pulp	2.07	0.45
Periodontal ligament	0.689	0.45
Medullary bone	1.370	0.3
Cortical bone	13.700	0.3
Aligner	528	0.36
Attachments	12,500	0.36
Bonded buttons	206,000	0.3
Extralveolar screw	103,400	0.35

Abbreviations: MPa, megapascal; v, resulting Poisson's; *Z*, *Z* axis.

**FIGURE 2:**
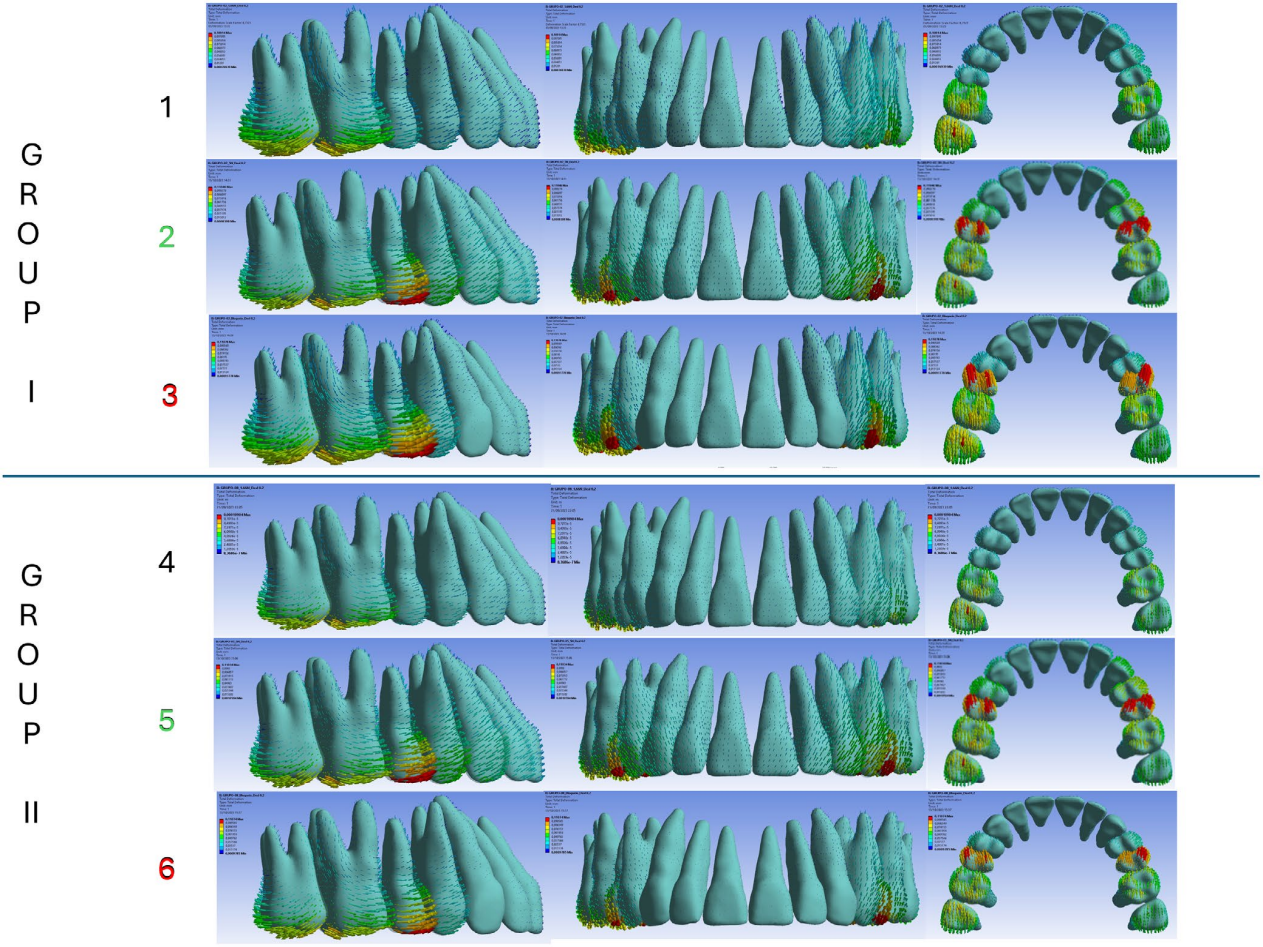
Initial tooth displacement (in mm) and stress (in MPa) of maxillary permanent teeth with 0.2 mm of distal activation. Groups I and II represent aligners without and with attachments, respectively. The different coloured numbers indicate the type of distal activation and skeletal anchorage: Numbers 1 and 4 (black): Distal activation of the second molars only, with 1.66 N of active skeletal anchorage. Numbers 2 and 5 (green): Distal activation of both first and second molars, with 1.66 N of active skeletal anchorage. Numbers 3 and 6 (red): Distal activation of both molars with passive skeletal anchorage.

Each model contained an average of 753,406 nodes and 472,591 elements. Data analysis compared quantitative values from the models with results from predefined areas of interest. The modified von Mises criterion was applied to calculate and present maximum tensile and minimum compressive stresses in megapascals (MPa). Displacements were measured at the mesiobuccal cusp tip of the second molar, the canine cusp tip, and the incisal edge of the upper central incisor to assess the immediate initial orthodontic movement during upper second molar distalization. Vector arrows indicated both the direction and magnitude of displacement in the coronal (*x*‐), sagittal (*y*‐), and vertical (*z*‐) planes, and values were reported in millimetres (mm) (Figures 1 and 2; Table 2).

**TABLE 2:** Initial displacement tendency (mm) of the upper right second molars, canines, and central incisors of each model (1–6), according to axes *X*, *Y*, and *Z*.

Anchorage force (N)	Attachments	Distalization activation force (0.2 mm)	Second molars			Canines			Central incisors		
			*X*	*Y*	*Z*	*X*	*Y*	*Z*	*X*	*Y*	*Z*
1.66 N from skeletal anchorage to the button	None	Between first and second molars	0.34e‐2	5.7e‐2	0.1e‐2	2.1e‐2	‐1.2e‐2	2.1e‐2	1e‐2	−0.4e‐2	0.9e‐2
1.66 N from skeletal anchorage to the button	None	Between first and second molars, and first molars and second premolars	0.32e‐2	5.59e‐2	0.09e‐2	3.22e‐2	−3.17e‐2	3.36e‐2	1.19e‐2	−1.37e‐2	1.65e‐2
0.12″ stainless steel ligature from skeletal anchorage to the button	None	Between first and second molars, and first molars and second premolars	0.35e‐2	5.6e‐2	0.042e‐2	0.1e‐2	0.82e‐2	0.71e‐2	0.36e‐2	−0.34e‐2	0.48e‐2
1.66 N from skeletal anchorage to the button	Vertical	Between first and second molars	0.3e‐2	5.7e‐2	0.1e‐2	2.2e‐2	−1.4e‐2	2.1e‐2	1e‐2	−0.5e‐2	0.9e‐2
1.66 N from skeletal anchorage to the button	Vertical	Between first and second molars, and first molars and second premolars	0.31e‐2	5.52e‐2	0.09e‐2	3.29e‐2	−3.42e‐2	3.47e‐2	1.18e‐2	−1.5e‐2	1.72e‐2
0.12″ stainless steel ligature from skeletal anchorage to the button	Vertical	Between first and second molars, and first molars second premolars	0.35e‐2	5.6e‐2	0.047e‐2	0.11e‐2	0.77e‐2	0.72e‐2	0.35e‐2	−0.39e‐2	0.49e‐2

## 3 Results

**FIGURE 3:**
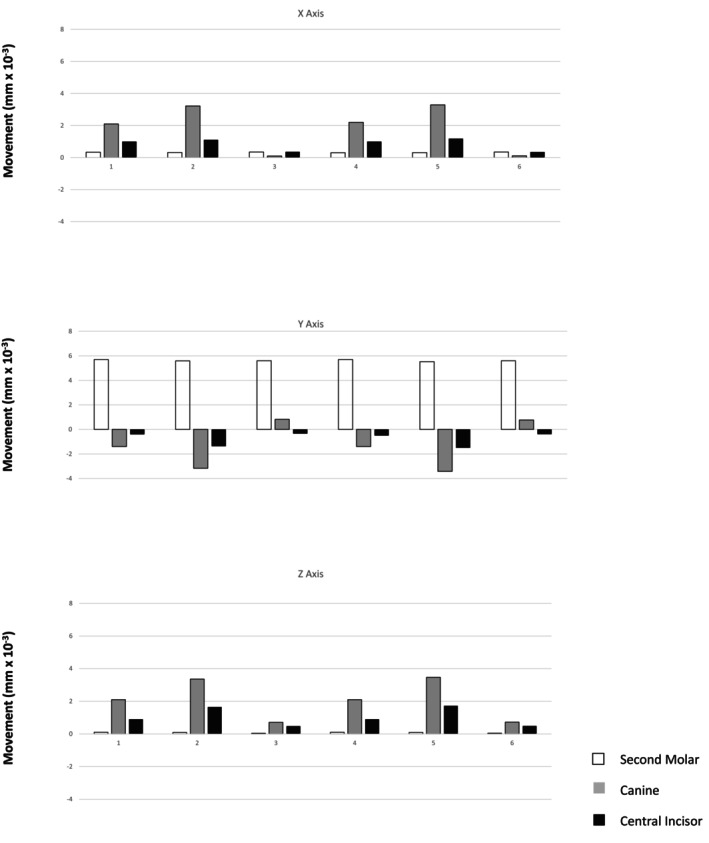
Immediate displacement of the second molars, canines, and upper central incisors along the *x*‐axis (positive values represent displacement toward the mid‐sagittal plane; negative values represent displacement away from it), the *y*‐axis (positive values represent distal displacement; negative values represent mesial displacement), and the *z*‐axis (positive values represent intrusive displacement; negative values represent extrusive displacement).

The results are presented as follows: initial displacement in millimetres (mm) (Table 2); modified von Mises stress in MPa (Figure 2); movement tendencies of the upper second molar, canine, and central incisor in all spatial planes (Figure 3); and the percentage of second molar distalization and initial anchorage loss of the canine and central incisor in each model (Table 3). This percentage was calculated by dividing the displacement of each tooth by the absolute sum of the displacements of the second molar, canine, and central incisor.

**TABLE 3:** Ratio (%) of upper second molar distal movement to canine and central incisor anchorage loss, with second‐first molars (Models 1 and 4) or second‐first molars and first molar‐second premolar (Models 2, 3, 5 and 6) activation.

Attachment	Model	Upper tooth movement (%)			Anchorage loss (%)
		Second molar	Canine	Central incisor	Canine and central incisor
Absent	1	+78.08%	−16.43%	−5.48%	−21%
	2	+55.18%	−31.29%	−13.52%	44.81%
	3	+94.28%	+13.8%	−5.7%	−5.7%
Vertical	4	+75%	−18.42%	−6.58%	−25%
	5	+52.87%	−32.76%	−14.37%	−47.13%
	6	+93.49%	+12.85%	−6.51%	−6.51%

*Note:* (+) distal movement; (−) Anchorage loss; (%) the percentage of each tooth movement in relation to the absolute sum of the second molar, canine and central incisor movements.

The *x*‐axis represents the mid‐sagittal plane. Positive values indicate movement toward the midline (contraction), whereas negative values indicate movement away from the midline (expansion). In all models, teeth generally shifted toward the midline. The second molars exhibited consistent, minimal displacement across all setups. In contrast, canines demonstrated the most pronounced initial movement, followed by central incisors. This pattern was observed regardless of the presence of vertical attachments on molars and premolars (Figure 3; Table 2).

With vertical attachments and active anchorage (1.66 N; Models 4 and 5), canine displacement toward the midline increased. In contrast, movement of the second molars and central incisors was similar to that observed in models without attachments but with the same active anchorage (Models 1 and 4) (Figure 3; Table 2). In models with attachments and passive anchorage (Models 3 and 6), canine and incisor displacement was markedly reduced—particularly in the upper canines, where movement was nearly eliminated (Figure 3; Table 2).

The *y*‐axis represents anteroposterior displacement; positive values indicate distal movement, and negative values indicate mesial movement. Immediate distal displacement of the upper second molars occurred across all models, regardless of anchorage type or 0.2 mm activation location (molar–molar or molar–premolar) (Figures 2 and 3; Tables 2 and 3). In active anchorage models (1.66 N) without attachments, second molar distal movement ranged from 55.18% (Model 2) to 78.08% (Model 1). With vertical attachments, this range was 52.87% (Model 5) to 75% (Model 4). Notably, when activation included the second premolars, the molar movement‐to‐anchorage loss ratio decreased.

In contrast, passive anchorage using a 0.012‐in. stainless steel ligature yielded the most favourable results. Without attachments (Model 3), second molar movement accounted for 94.28% of total displacement; with attachments (Model 6), it accounted for 93.49% (Figures 2 and 3; Tables 2 and 3). Thus, passive anchorage—regardless of attachment use—achieved the highest molar‐to‐anchorage ratios. A distinct pattern emerged in Models 3 and 6: the canines followed the molars distally, representing 13.80% and 12.85% of displacement, respectively—an inversion compared with the other models. Only the central incisors continued to exhibit anchorage loss, although to a lesser degree (5.7% in Model 3 and 6.51% in Model 6). The least favourable result (52.87%) occurred in Model 5, which combined active anchorage, vertical attachments, and premolar activation (Figures 2 and 3; Tables 2 and 3).

For the *z*‐axis (vertical displacement/intrusion), immediate displacement trends were consistent across all models, although magnitudes varied (Figure 3; Table 2). Intrusive displacement was minimal in the second molars, slightly greater in the central incisors, and greatest in the canines. As with the *x*‐axis, vertical displacement was greater in models using active anchorage with vertical attachments (Figure 3; Table 2).

Conversely, models with passive anchorage via ligature wire—without attachments (Model 3) and with attachments (Model 6)—showed markedly reduced vertical displacement of canines and central incisors (Figures 2 and 3; Table 2). These results indicate that passive anchorage more effectively limits unwanted vertical movement, regardless of attachment use.

## 4 Discussion

The expectation that clear aligners alone can achieve molar distalization without affecting anterior teeth has not been met. Various adjunctive strategies have been proposed to minimise these effects, including the use of Class II elastics [16, 17], differential sequencing of tooth movements [4, 18, 20, 21, 22], variations in attachment configuration [10, 12, 21], and skeletal anchorage [4, 6, 8, 12, 21, 23]. Among these, TADs have demonstrated superior control in stabilising anchorage during molar distalization [4, 11].

Nevertheless, key biomechanical questions remain: Is there a difference in outcome between applying active force (e.g., elastics) and passive anchorage (e.g., ligature wire)? Do vertical rectangular attachments influence the results? How does applying distal force solely to the second molars compare with also activating the first molars via the second premolars? This study employed FEA to simulate and compare these clinical scenarios [22]. All six models exhibited similar immediate distal displacement of the upper second molars (Figures 2 and 3; Tables 2 and 3), supporting the effectiveness of each mechanical setup. However, the impact on adjacent teeth varied. The distal molar movement‐to‐anchorage loss ratio—particularly along the *y*‐axis (mesiodistal)—proved to be a critical metric. Passive anchorage consistently produced better results, with ratios exceeding 90%, whereas active anchorage averaged 65.28% (range: 52.87%–78.08%). These findings contrast with those of Liu et al. [5], who reported improved molar displacement with increased anchorage force but also noted greater side effects.

Notably, models with passive anchorage and activation of both the first and second molars (Models 3 and 6) outperformed those with active anchorage limited to the second molars (Models 1 and 4). For example, passive anchorage without attachments (Model 3) achieved a 94.28% distal molar movement‐to‐anchorage loss ratio, whereas active anchorage with molar–premolar activation (Model 2) achieved only 55.18%. Similarly, with non‐bevelled vertical attachments, passive anchorage (Model 6) produced a 93.49% ratio compared with 52.87% for active anchorage (Model 5). These comparisons suggest that vertical attachments have minimal impact when comparing analogous models, consistent with previous findings for upper molar distalization using FEA [21, 22]. One possible explanation is that continuous active forces from skeletal anchorage to bonded buttons may induce unintended movement of the anchor teeth. The aligner may be unable to fully counteract these forces and moments, leading to tracking loss and unplanned displacements—even at sites distant from the point of force application.

A novel finding of this study—one not previously reported—is that with passive anchorage, both with and without attachments, the upper canines followed the distal displacement of the second molars (Tables 2 and 3). Clinically, this outcome is desirable because it reflects cooperative rather than antagonistic movement between the posterior and anterior segments. These results support the hypothesis that passive anchorage, without continuous extraneous force, maintains the intended biomechanical integrity of the aligner.

Regarding the *x*‐axis (transverse dimension), displacement toward the mid‐sagittal plane was generally observed, particularly in canines, followed by central incisors, across all models. Active anchorage with vertical attachments intensified this effect, whereas second molar and central incisor displacement remained minimal (Figure 3; Table 2). Notably, passive anchorage nearly eliminated transverse displacement—especially in canines—underscoring its stabilising effect in the buccolingual direction.

In the vertical dimension (*z*‐axis), all models exhibited minor intrusion of the second molars, with greater intrusion observed in the central incisors and canines (Figure 3; Table 2). Non‐bevelled vertical attachments had little effect on vertical displacement. The most pronounced intrusion occurred in models with active anchorage extending to the second premolars (Models 2 and 5). Conversely, passive anchorage (Models 3 and 6) substantially reduced vertical displacement of the canines and incisors, regardless of attachment presence (Figure 3; Table 2).

In summary, these findings strongly suggest that passive skeletal anchorage better preserves the intended biomechanics during molar distalization with clear aligners compared with active methods. Rather than increasing anchorage force—which may compromise aligner fit and lead to undesired tooth movement—using a ligature wire from the skeletal anchorage device to a canine or premolar appears more effective, allowing the aligner to guide tooth movements as designed. This study provides strong evidence supporting passive force delivery, specifically using ligature wires to anterior teeth, to maintain aligner integrity and achieve more predictable molar distalization.

The limitations of this study must be acknowledged, as it was based on initial tooth movement simulated by FEA. As a laboratory‐based investigation, it did not account for intraoral variables such as occlusal forces, saliva, patient compliance, and variations in periodontal condition. The CBCT data were obtained from a young adult with a well‐proportioned facial pattern. Consequently, these findings should be interpreted with caution when extrapolated to individuals with dolichofacial or brachyfacial skeletal types or those presenting with a Class II maxillomandibular discrepancy requiring maxillary posterior dental distalization. Moreover, the potential effects of attachments on the maxillary canines and the possible displacement of root apices of the evaluated teeth were not assessed and warrant future investigation. Therefore, additional clinical studies—preferably randomised controlled trials—are needed to validate these biomechanical approaches for maxillary molar distalization.

## 5 Conclusions

Ligature‐based passive anchorage produced markedly higher molar movement‐to‐anchorage loss ratios (exceeding 90%) compared with elastic‐based active anchorage systems (approximately 65%), effectively minimising undesirable tooth movements.

The presence or absence of vertical attachments resulted in comparable outcomes across analogous models, indicating that attachments do not substantially affect anchorage control during molar distalization.

## Author Contributions

**Weber Jose da Silva Ursi:** investigation, data curation, writing – original draft. **Yamyle Claudia Velásquez Barragán:** resources, methodology, investigation, data curation. **Ki Beom Kim:** formal analysis, writing – review and editing. **Carlos Flores‐Mir:** formal analysis, writing – review and editing. **Guilherme de Araujo Almeida:** resources, conceptualization, methodology, data curation, writing – original draft, writing – review and editing, supervision.

## Ethics Statement

The present study was approved by the Ethical Committee of the Federal University of Uberlandia (UFU), Brazil, and the National Research Ethics Council (CONEP‐Brazil) (approval code: 68334822.0.0000.5152).

## Consent

Written informed consent was obtained from the study participant.

## Conflicts of Interest

The authors declare no conflicts of interest.

## Data Availability

The data that support the findings of this article are available from the corresponding author (GAA) upon reasonable request.

## References

[1] E. Krieger , J. Seiferth , I. Marinello , et al., “Invisalign Treatment in the Anterior Region: Were the Predicted Tooth Movements Achieved?,” Journal of Orofacial Orthopedics 73, no. 5 (2012): 365–376. 22890691 10.1007/s00056-012-0097-9

[2] E. H. Sung , S. J. Kim , Y. S. Chun , Y. C. Park , H. S. Yu , and K. J. Lee , “Distalization Pattern of Whole Maxillary Dentition According to Force Application Points,” Korean Journal of Orthodontics 45, no. 1 (2015): 20–28. 25667914 10.4041/kjod.2015.45.1.20 PMC4320314

[3] X. Li , Y. Xu , Y. Wang , and J. Wang , “Investigating the Anatomical Relationship Between the Maxillary Molars and Sinus Floor in a Chinese Population Using Cone‐Beam Computed Tomography,” BMC Oral Health 19, no. 1 (2019): 282. 31842859 10.1186/s12903-019-0969-0 PMC6915992

[4] R. H. d. C. Grec , G. Janson , N. C. Branco , P. G. Moura‐Grec , M. P. Patel , and J. F. Castanha Henriques , “Intraoral Distalizer Effects With Conventional and Skeletal Anchorage: A Meta‐Analysis,” American Journal of Orthodontics and Dentofacial Orthopedics 143, no. 5 (2013): 602–615. 23631962 10.1016/j.ajodo.2012.11.024

[5] X. Liu , J. Wu , Y. Cheng , et al., “Effective Contribution Ratio of the Molar During Sequential Distalization Using Clear Aligners and Micro‐Implant Anchorage: A Finite Element Study,” Progress in Orthodontics 24, no. 1 (2023): 35. 37806991 10.1186/s40510-023-00485-0 PMC10560653

[6] H. Gao , L. Luo , and J. Liu , “Three‐Dimensional Finite Element Analysis of Maxillary Molar Distalization Treated With Clear Aligners Combined With Different Traction Methods,” Progress in Orthodontics 25, no. 1 (2024): 47. 39648191 10.1186/s40510-024-00546-y PMC11625702

[7] J. Cui , C. Yao , Z. Zhang , et al., “Maxillary Molar Distalization Treated With Clear Aligners Combined With Mini‐Implants and Angel Button Using Different Traction Force: A Finite Element Study,” Computer Methods in Biomechanics and Biomedical Engineering 27, no. 3 (2024): 296–305. 36939836 10.1080/10255842.2023.2183735

[8] M. Matias , C. Flores‐Mir , M. R. de Almeida , et al., “Miniscrew Insertion Sites of Infrazygomatic Crest and Mandibular Buccal Shelf in Different Vertical Craniofacial Patterns: A Cone‐Beam Computed Tomography Study,” Korean Journal of Orthodontics 51, no. 6 (2021): 387–396. 34803027 10.4041/kjod.2021.51.6.387 PMC8607118

[9] L. Ji , B. Li , and X. Wu , “Evaluation of Biomechanics Using Different Traction Devices in Distalization of Maxillary Molar With Clear Aligners: A Finite Element Study,” Computer Methods in Biomechanics and Biomedical Engineering 26, no. 5 (2023): 559–567. 35543236 10.1080/10255842.2022.2073789

[10] A. Inan and M. Gonca , “Effects of Aligner Activation and Power Arm Length and Material on Canine Displacement and Periodontal Ligament Stress: A Finite Element Analysis,” Progress in Orthodontics 24, no. 1 (2023): 40. 38008884 10.1186/s40510-023-00492-1 PMC10678869

[11] L. Jia , C. Wang , L. Li , et al., “The Effects of Lingual Buttons, Precision Cuts, and Patient‐Specific Attachments During Maxillary Molar Distalization With Clear Aligners: Comparison of Finite Element Analysis,” American Journal of Orthodontics and Dentofacial Orthopedics 163, no. 1 (2023): e1–e12. 36435687 10.1016/j.ajodo.2022.10.010

[12] Z. Guo , R. Zhang , C. Guo , X. Li , Z. Jin , and Q. Liu , “A Retrospective Study of Alveolar Bone Remodelling After Anterior Retraction in Orthodontic Tooth Extraction Cases With Clear Aligners and Fixed Appliances,” Orthodontics & Craniofacial Research 27, no. 2 (2024): 220–227. 37578004 10.1111/ocr.12705

[13] X. Chen , Y. Shi , J. Yuan , Y. Li , and W. Chen , “Factors Influencing the Efficacy of Invisalign in Molar Distalization and Tooth Movement,” Frontiers in Bioengineering and Biotechnology 11 (2023): 1215169. 37954021 10.3389/fbioe.2023.1215169 PMC10634526

[14] S. V. N. Jaecques , H. Van Oosterwyck , L. Muraru , et al., “Individualised, Micro CT‐Based Finite Element Modelling as a Tool for Biomechanical Analysis Related to Tissue Engineering of Bone,” Biomaterials 25, no. 9 (2004): 1683–1696. 14697870 10.1016/s0142-9612(03)00516-7

[15] J. P. Gomez , F. M. Peña , V. Martínez , D. C. Giraldo , and C. I. Cardona , “Initial Force Systems During Bodily Tooth Movement With Plastic Aligners and Composite Attachments: A Three‐Dimensional Finite Element Analysis,” Angle Orthodontist 85, no. 3 (2015): 454–460. 25181252 10.2319/050714-330.1 PMC8612436

[16] P. Soares , A. Machado , L. Zeola , et al., “Loading and Composite Restoration Assessment of Various Non‐Carious Cervical Lesions Morphologies – 3D Finite Element Analysis,” Australian Dental Journal 60, no. 3 (2015): 309–316. 25312697 10.1111/adj.12233

[17] X. Liu , Y. Cheng , W. Qin , et al., “Effects of Upper‐Molar Distalization Using Clear Aligners in Combination With Class II Elastics: A Three‐Dimensional Finite Element Analysis,” BMC Oral Health 22, no. 1 (2022): 546. 36456944 10.1186/s12903-022-02526-2 PMC9714146

[18] S. Loberto , V. Paoloni , C. Pavoni , P. Cozza , and R. Lione , “Anchorage Loss Evaluation During Maxillary Molars Distalization Performed by Clear Aligners: A Retrospective Study on 3D Digital Casts,” Applied Sciences 13, no. 6 (2023): 3646.

[19] D. G. Garib , F. Miranda , M. S. Yatabe , et al., “Superimposition of Maxillary Digital Models Using the Palatal Rugae: Does Ageing Affect the Reliability?,” American Journal of Orthodontics and Dentofacial Orthopedics 22, no. 3 (2019): 183–193. 10.1111/ocr.12309 PMC6642031 30844126

[20] S. Ravera , T. Castroflorio , F. Garino , S. Daher , G. Cugliari , and A. Deregibus , “Maxillary Molar Distalization With Aligners in Adult Patients: A Multicenter Retrospective Study,” Progress in Orthodontics 17, no. 1 (2016): 12. 27041551 10.1186/s40510-016-0126-0 PMC4834290

[21] B. Mao , Y. Tian , Y. Xiao , et al., “Effect of Different Anchorage Reinforcement Methods on Long‐Term Maxillary Whole Arch Distalization With Clear Aligner: A 4D Finite Element Study With Staging Simulation,” Bioengineering (Basel) 11, no. 1 (2024): 3. 10.3390/bioengineering11010003 PMC10813679 38275571

[22] C. Ayidağa and B. Kamiloğlu , “Effects of Variable Composite Attachment Shapes in Controlling Upper Molar Distalization With Aligners: A Nonlinear Finite Element Study,” Journal of Healthcare Engineering 2021 (2021): 5557483. 34457219 10.1155/2021/5557483 PMC8397573

[23] J. Li , Y. Yang , Z. Tang , et al., “Biomechanical Analysis of the Effect of Class II Traction Configurations and Aligner Overtreatment on Molar Distalization: A Finite‐Element Study,” European Journal of Orthodontics 46, no. 6 (2024): cjae055. 39412181 10.1093/ejo/cjae055

